# Regime Shifts in the Anthropocene: Drivers, Risks, and Resilience

**DOI:** 10.1371/journal.pone.0134639

**Published:** 2015-08-12

**Authors:** Juan Carlos Rocha, Garry D. Peterson, Reinette Biggs

**Affiliations:** 1 Stockholm Resilience Centre, Stockholm University, Kräftriket 2B, 10691, Stockholm, Sweden; 2 Centre for Studies in Complexity, Stellenbosch University, Private Bag X1, Matieland, 7602, Stellenbosch, South Africa; Seagrass Ecosystem Research Group, Swansea University, UNITED KINGDOM

## Abstract

Many ecosystems can experience regime shifts: surprising, large and persistent changes in the function and structure of ecosystems. Assessing whether continued global change will lead to further regime shifts, or has the potential to trigger cascading regime shifts has been a central question in global change policy. Addressing this issue has, however, been hampered by the focus of regime shift research on specific cases and types of regime shifts. To systematically assess the global risk of regime shifts we conducted a comparative analysis of 25 generic types of regime shifts across marine, terrestrial and polar systems; identifying their drivers, and impacts on ecosystem services. Our results show that the drivers of regime shifts are diverse and co-occur strongly, which suggests that continued global change can be expected to synchronously increase the risk of multiple regime shifts. Furthermore, many regime shift drivers are related to climate change and food production, whose links to the continued expansion of human activities makes them difficult to limit. Because many regime shifts can amplify the drivers of other regime shifts, continued global change can also be expected to increase the risk of cascading regime shifts. Nevertheless, the variety of scales at which regime shift drivers operate provides opportunities for reducing the risk of many types of regime shifts by addressing local or regional drivers, even in the absence of rapid reduction of global drivers.

## Introduction

We are living in the Anthropocene, an epoch where human actions intentionally and accidentally are changing planetary processes [1–5] and ecosystems [6]. While some of these changes have been gradual, others have led to surprising, large and persistent ecological regime shifts [7,8]. Such shifts challenge ecological management and governance because they substantially alter the availability of ecosystems services [9], while being difficult to predict and reverse [7]. While the importance of ecological regime shifts is increasingly recognized [3,10–12], the variety of regime shifts and their drivers is less well known. Only a handful of studies have compared multiple regime shifts, but have always focused on a specific system, such as the climate system [3], or agricultural [11], terrestrial Arctic [13], and marine ecosystems [14]. A few reviews have synthesized the drivers of regime shifts, for instance in coral reefs [15] and drylands [16]. However, a global comparison of drivers of regime shifts across different systems types has never before been undertaken.

Most drivers of global change are increasing along with an exponential growth of the world’s economy [4,6,17], and the frequency and intensity of regime shifts are expected to increase too [18]. However most research on regime shifts is ill-suited to examine this proposition. Research on regime shifts has typically focused on theoretical models [8,19,20], empirical evidence of regime shifts[21], or potential early warning signals [12,22]. These approaches require in-depth knowledge of the causal structure of the system or high-quality temporal data, leading to a focus on the analysis of particular cases of regime shifts. Here we complement this work by synthesizing and comparing different types of regime shifts in terms of global change impacts and opportunities for management. Our aim is to understand: i) What are the main drivers of regime shifts globally? ii) What are their most common impacts on ecosystem services? And, iii) what can be done to manage or avoid them?

## Materials and Methods

We addressed these questions using a diverse set of methods in a six-phase process. First we developed a framework for data collection that facilitates comparison among regime shifts, namely the regime shifts database. Second, we identified and grouped the different drivers into hierarchical classes, distinguishing direct from indirect drivers. Third, strategies to manage regime shift drivers were identified and classified according to the scale at which action needs to be taken to tackle the effect of each driver. Fourth, to better understand the relative importance of drivers, we studied their patterns of co-occurrence by constructing and simulating networks. Fifth, to discover what factors explained patterns among regime shifts and their drivers, exponential random graph models were used to explore what types of local interactions were consistent with the observed global patterns of the network. Sixth, to identify the most common impacts on ecosystem services, or the most common interactions among driver types, we analyzed the drivers and regime shifts datasets using ordering methods. Each of these steps are described in the following sections.

### Data

The regime shift database (RSDB) was created to synthesize, compare and share scientific knowledge about regime shifts in social-ecological systems [www.regimeshifts.org]. The RSDB currently provides a synthesis of >800 scientific papers, summarizing over 200 cases and about 25 generic types of regime shifts [23]. It presents information in both plain text and 92 categorical variables about the i) main drivers of change, ii) impacts on ecosystem services, ecosystem processes and human well-being, iii) land use, ecosystem type and spatial-temporal scale at which each regime shift typically occurs, iv) possible managerial options, and v) assessment of the reversibility of the regime shift and the level of uncertainty related to the existence of the regime shift, and its underlying mechanism. The review of each regime shift is available online and wherever possible each entry has been written or peer-reviewed by an expert on the topic.

The database collects the most studied types of regime shifts in social-ecological systems [10]. Examples of regime shifts include i) well-established cases like eutrophication [21], where lakes turn from clear water to murky water leading to reduced fishing productivity and toxic algae blooms; ii) controversial cases like dryland degradation when dry forest and savanna shift to deserts and bare soils, significantly reducing ecosystem services such as agricultural production and water cycling [16]; and iii) proposed shifts like the collapse of the Greenland ice sheet where the frequency and intensity of warm events will shift the ice sheet from permanent to occasional, reducing services such as coast line protection and climate regulation [24]. An overview of the 25 regime shifts analysed in this paper is given in Table 1.

**Table 1:** Summary of the 25 regime shifts examples from the regime shifts database used in this analysis.

Regime Shift	Initial regime	Alternative regime	Ecosystem	Ecosystem Services affected *	Selected drivers	Key reference
Eutrophication	Clear water	Murky water	Aquatic—Coastal	Fisheries, water purification,recreation	Nutrient inputs, agriculture, urban storm water runoff	[21]
Marine food web simplification	Predators dominated	Lower trophic groups dominated	Aquatic—Coastal	Fisheries, pest & disease regulation, recreation	Climate change, nutrient inputs, fishing	[50]
Hypoxia	Normoxia	Hypoxia, anoxia	Aquatic—Coastal	Fisheries, pest & disease regulation, recreation	Fertilizers use, upwellings, water stratification	[51]
Fisheries collapse	High abundance of commercial fish	Low abundance of commercial fish	Aquatic—Marine	Fisheries, pest & disease regulation, biodiversity	Fishing, NSO like events, upwellings	[52]
Floating plants	Submerged plants dominance	Floating plants dominance	Aquatic	Fisheries, pest & disease regulation, recreation	Fertilizers use, sediments, sewage	[53]
River channel change	Old channel course	New channel regime	Aquatic	Freshwater, food production, regulation soil erosion, transport	Erosion, floods, rainfall variability	[54]
Mangroves transitions	Mangrove forest	Salt marshes, rocky tidal or shrimp farms	Aquatic—coastal	Fisheries, timber, regulation soil erosion, recreation	Deforestation, coastal erosion, sea level rise	[55]
Sea grass transitions	Sea grass dominated	Algae dominated or bare sediments	Aquatic—coastal	Fisheries, water purification, regulation soil erosion	Sediments, aquaculture, fishing	[56]
Marine eutrophication	Clear water	Nutrient rich water	Marine	Fisheries, water purification, recreation	Nutrient inputs, climate change, sewage	[57]
West Antarctica Ice Sheet collapse	Full glacial or modern interglacial	Extreme interglacial	Polar	Climate regulation, natural hazards protection	Climate change, sea surface temperature, upwelling	[58]
Bivalves collapse	High abundance of bivalves	Low abundance of bivalves	Marine	Water purification, fisheries, biodiversity	Aquaculture, disease, sediments	[59]
Coral transitions	Coral dominated reefs	Macro-algae, soft corals, corallimorpharians, sponges, or urchin barrens	Marine	Biodiversity, fisheries, recreation, coastal protection	Fishing, climate change, ocean acidification	[60]
Kelp transitions	Canopy forming algae	Turf forming algae, urchin barrens	Marine	Fishing, biodiversity, recreation	ENSO like events, fishing, nutrient inputs	[61]
Encroachment	Grass dominated savanna	Shrub dominated savanna	Savannas	Livestock, climate regulation, biodiversity	Ranching (livestock), irrigation, fire frequency	[62]
Soil salinization	Low salinity soils	High salinity soils	Dry lands	Fresh water, food production, soil erosion regulation, biodiversity	Agriculture, irrigation, floods	[63]
Forest to savannas	Forest	Savanna	Forest—Savanna	Biodiversity, climate regulation, water cycling, food production	Deforestation, fire frequency, droughts	[64]
Dry land degradation	Dry lands: savannas, dry forest	Deserts	Dry lands	Freshwater, food production, timber and fuel, climate regulation, water regulation	Erosion, droughts, water infrastructure	[16]
Tundra to forest	Tundra	Forest	Tundra	Livestock, wildlife food, climate regulation, timber	Climate change, hunting, ranching (livestock)	[65]
Monsoon	Strong monsoon	Weak monsoon	Marine—Terrestrial	Water cycling, food production, timber, climate regulation	Deforestation, droughts, sea surface temperature	[66]
Peatlands	Low productivity & high C accumulation	High productivity & low C accumulation	Peatlands	Nutrient cycling (C), climate regulation	Nutrient intputs, precipitation, wetland drainage	[67]
Greenland Ice Sheet melting	Permanent ice sheet	No permanent ice sheet	Polar	Coastline protection, climate regulation, water regulation	Climate change, green house gases, water stratification	[68]
Thermohaline Circulation Collapse	Strong thermohaline circulation	Collapse of thermohaline circulation	Polar—Marine	Climate regulation, biodiversity, food production	Sea surface temperature, sea water density, climate change	[69]
Salt marshes to tidal flats	Salt marshes	Tidal or subtidal flat	Marine—coastal	Pollution filtration, storm protection, fisheries, food production.	Coastal erosion, nutrient inputs, sea level rise	[70]
Arctic Sea Ice collapse	Arctic with summer ice	Arctic without summer ice	Polar	Climate regulation, aesthetic values, natural hazards protection	Climate change, green house gases, sea surface temperature	[71]
Steppe to tundra	Steppe	Tundra	Steppe	Biodiversity, food production, climate regulation	Climate change, temperature, hunting	[72]

*Only the main ecosystem service impacts, a selected list of drivers and a key reference are shown. For an extended review please check www.regimeshifts.org.

### Driver identification

Drivers include natural or human induced changes that have been identified as directly or indirectly producing a regime shift [6,25]. We first collected a preliminary list of drivers for each regime shift taking as a starting point that it should be referenced in the academic literature that the variable has causal influence on the regime shift. For each regime shift we draw a causal loop diagram, a graphical representation of the causal structure of the system [26]. References and descriptions of each driver plus causal diagrams are available in the RSDB. To avoid ambiguities and conflicting definitions across different scholars, we defined drivers as variables outside the feedback mechanisms of the system, thus they are variables independent of the dynamics of the system. Direct drivers are those that influence the internal processes or feedbacks underlying a regime shift, and indirect drivers those that alter one or more direct drivers [25]. Based on the minimum distance to a feedback loop, we assessed the directedness of a driver as the shortest number of steps of separation to the feedbacks. This classification was done for each regime shift, therefore when comparing regime shifts a driver in one system can be part of an feedback in another.

To enable consistent comparison of drivers we systematically ensured that drivers were defined consistently across the database. After the first identification of drivers we checked for semantic cohesion, to avoid different words referring to the same driver. So for example cropping and agriculture were renamed agriculture. When the variables explicitly referred to different phenomena, different names were kept. For example rainfall variability and precipitation were kept separately as the first refers to variability and the second to total quantity. We further classified drivers as belonging to different types of global change by slightly modifying previous classifications [10,25]. We identified 15 detailed categories of drivers, which were further grouped into 5 broad categories: *habitat modification*, *food production*, *nutrients and pollutants*, *resource extraction* and *spill-over effects*. Thus, we distinguish between drivers stemming directly from human activities (e.g. fertilizer use) and drivers affected by the knock-on or ‘spill-over’ effects of these activities on natural processes (e.g. sedimentation or upwelling). A worked example is presented in S1 File.

### Scale of management

To examine management options for drivers of regime shifts we classified each driver by the scale it could be managed. Managerial options for each regime shift are synthesized in the RSDB. We exclusively classified each driver as requiring management at either local, national, or international scales. We considered a driver to be local if it could be mitigated substantially by changes made at the landscape or municipality level. If changes at the watershed or regional level could strongly counteract a driver we classified it as regional to national, and if actions to influence a driver require global or continental coordination we coded it as international. For drivers that can be managed at more than one scale, we chose the broadest scale at which managerial actions are likely to be strong enough to avoid the shift, as the broader scale could subsume multiple smaller scale actions. To make management actions comparable, we calculated the proportion of drivers per regime shift (%) that belong to each management scale.

### Network simulations

To better understand the relative importance of regime shifts and drivers we constructed a bipartite network where a driver is connected to a regime shift if there is a reference in the academic literature that suggests causality or influence on its feedback mechanisms. The bipartite network was analysed by considering two network projections: a network of drivers connected by the regime shifts they caused, and a network of regime shifts connected by the drivers they share.

Since highly connected drivers are more likely to cause regime shifts and highly connected regime shifts are more vulnerable to different sets of drivers, the mean degree, the co-occurrence index and clustering coefficient [27,28] were measured and compared with 10000 random simulated networks. The mean degree is the average number of connections one would expect in a random node. The co-occurrence index measures how commonly two drivers or two regime shifts co-occur together, i.e.,how common open triangles are in the bipartite network. The clustering coefficient measures the ratio of closed 4 link paths (squares) over the number of open 3 link paths (open triangles), or whether closed micro structures (clusters) are more common than open structures.

We assume that the relative importance of a driver, or the number of times that is reported causing different regime shifts, depends on the particular sample of regime shifts being analyzed. To test whether the importance of different drivers differed significantly, we randomly reshuffled the associations between drivers and regime shifts, keeping the number of links per node unchanged. Simulations were performed in the R statistical software [29], using a Sequential Importance Sampling algorithm, in R’s networksis [30] and ergm [31] packages. The comparison between observed interactions and random data is fundamental to understanding whether the co-occurrence patterns we found are due to sampling noise or correspond to a real pattern. If the observed patterns deviate from random, there should be theoretical reasons why they diverge that we further explored with statistical modeling.

### Model fitting

Exponential random graph models [32] were used to explore what local processes could explain the emergent patterns in the networks. We tested whether certain minimal configurations are statistically more common (e.g. triangles) or if links are significantly more likely to occur if nodes share the same attribute (e.g. management scale). Nestedness [33] was calculated for the bipartite network to test if the generalist or idiosyncratic character of each driver in the network was related to its scale of management. We used the number of papers reported per regime shift on the ISI Web of Science by 2013 as an approximation of how extensively a regime shift has been studied.

To explore the processes underlying the network patterns, we modelled scale of management, nestedness, frequency and directedness as categorical variables or node covariates for drivers; while ecosystem type, nestedness, number of papers reported, and frequency were modelled as categorical variables or node covariates for regime shifts. The presence or absence of categorical variables in the RSDB was used to construct distance measures of how similar two regime shifts are depending on the variables shared. These distances were modelled as edge covariates for the regime shift network projection (see regime shifts clustering below). The bipartite network was modelled as binary network with geometrically weighted terms [34–36], while the one-mode projections were modelled following the specifications for weighted edges [37] and a Poisson distribution as reference. All models were fitted with ergm [31] and ergm.count [37] packages for R [29].

### Regime shifts and drivers clustering

We used multi-dimensional scaling to investigate the patterns underlying the clustering of regime shifts. First we calculated the Sorensen-Dice distance between regime shifts given the drivers they share. This measure favours the presence of common drivers in the network rather than their absence, and we use it because we are analyzing driver co-occurrence or regime shifts rather than straightforward difference among regime shifts. The hierarchical clustering was performed using the categorical variables of the RSDB after deleting zero columns, grouped by variables as follows: ecosystem processes (5 variables), provisioning services (8), regulating services (8), cultural services (4), drivers (10), land use (11), scales (8), and reversibility (3).

We analysed patterns among the drivers and the regime shifts in two ways. First, we used existing classifications [10,25] from global change research to classify drivers into 5 broad and 15 detailed categories (S1 Table). Applying matrix multiplication of the bipartite data to the drivers categorization, we obtained the number of drivers per regime shift that fall into each broad and detailed global change category. Second, we clustered the drivers based on patterns produced by their connections to regime shifts in the bipartite network. Jaccard distances measured in the bipartite matrix were used to organize the drivers into hierarchical clusters with an average method using the R package gplots [38]. These two approaches allowed us to compare how global change meta-drivers impact regime shifts, and to detect emergent patterns from our regime shift data based on the published literature.

## Results

**Fig 1:**
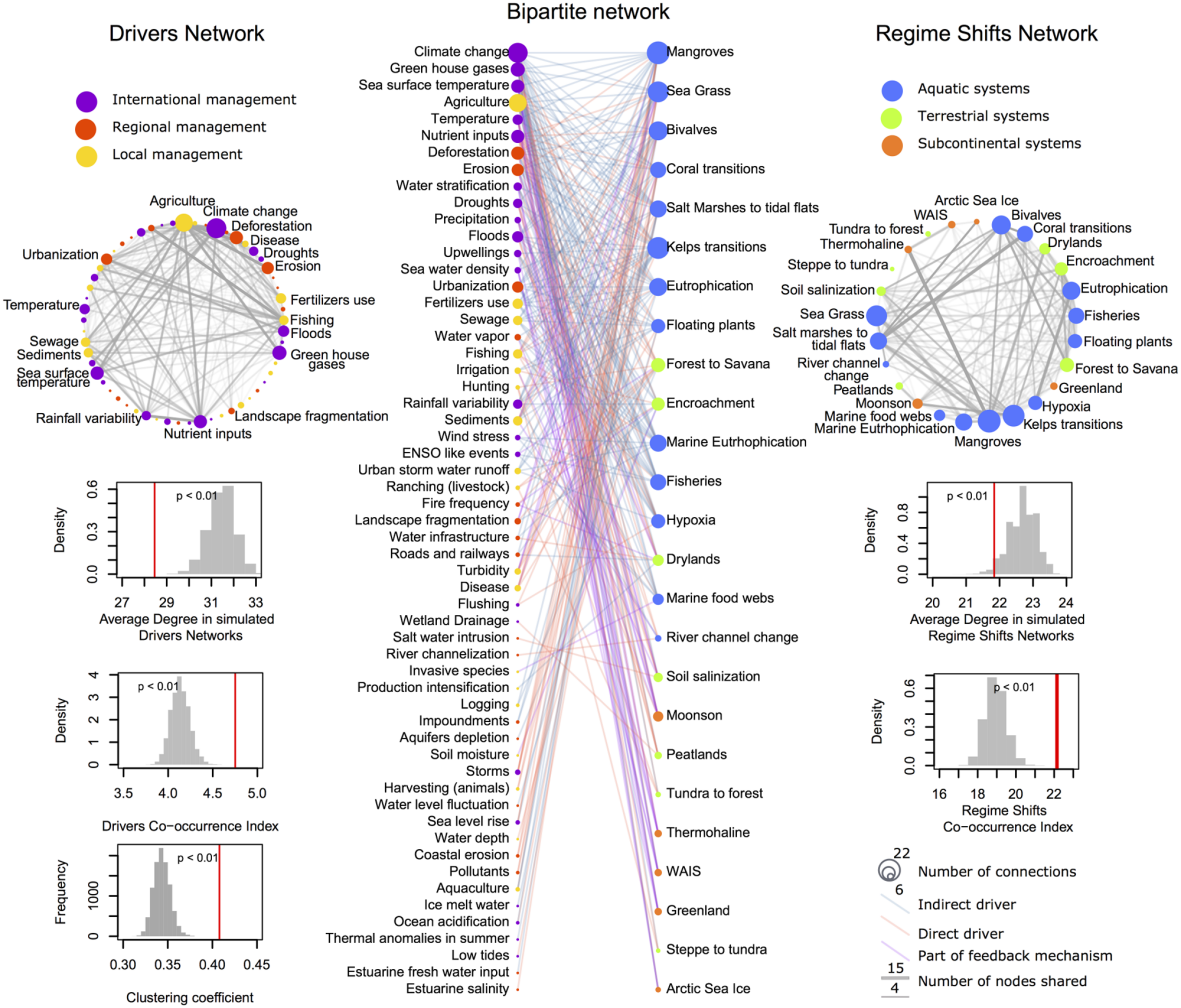
Regime shifts—Drivers Network. In the centre (A) the bipartite network of 57 drivers (left) and 25 regime shifts (right) organized by their nestedness. Highly nested nodes are idiosyncratic and are located on the lower part of the graph while nodes with low nesting are generalist and appear in the upper part. On the right (B) is the one-mode projection of regime shifts (N = 25). The width of the links is scaled by the number of drivers shared, while node size corresponds to the number of drivers per regime shift. On the left (C) is the one-mode projection of drivers (N = 57), with link width scaled by the number of regime shifts for which causality is shared, and node size proportional to the number of regime shifts per driver. Below each projection is the expected distributions for the co-occurrence index and average degree for the one-mode projection of the drivers and regime shifts networks. The bottom left panel shows the clustering coefficient for the bipartite network. For all structural statistics, the red lines mark the actual values for the observed data.

We identified 57 drivers underlying 25 regime shifts (Fig 1). The mean number of drivers per regime shift is 11.2, ranging from a low of 3 for *steppe to tundra* to a high of 22 for *mangrove collapse*. The most frequently reported drivers of regime shifts are *climate change*, *agriculture* and *fishing*, which are reported as drivers of 19, 17 and 15 regime shifts respectively (Fig 1). There are also 14 idiosyncratic drivers (~24%) that are unique to specific regime shifts. More than half of the connections between drivers and regime shifts are accounted for by 13 drivers (~22%). The most frequently co-occuring drivers, understood as the number of regime shifts they jointly drive, are *agriculture*, *climate change*, *nutrient inputs*, *deforestation*, *greenhouse gases*, *erosion* and *sea surface temperature*, where each pair occurs together in 10 or more regime shifts. The regime shifts with the greatest number of shared drivers are *marine eutrophication*, *sea grass collapse*, *fisheries collapse*, and *kelp transitions*, which have 8 drivers in common.

The regime shift-drivers network had a much higher clustering coefficient, higher co-occurrence index, and lower mean degree than randomized networks (t-test for all statistics P < 10−^15^, Fig 1). This result suggests that co-occurrence patterns among drivers are related to underlying processes. Furthermore, the network exhibits a nested structure: idiosyncratic drivers co-occur only with drivers that also co-occur with generalist ones (Fig 1 and S1 Fig). Surprisingly, the exponential random graph models show (S2 and S3 Tables) that the nested structure of the network is not due to global drivers being widely shared among regime shifts and local drivers being idiosyncratic. Rather, drivers that can be managed at local and regional scales are more likely to co-occur with drivers that can also be managed at the same scale. Drivers are significantly more likely to co-occur if they are indirect and generalist. Aquatic and subcontinental regime shifts tend to share the same set of drivers; while terrestrial and subcontinental regime shifts share fewer and more varied sets of drivers. Overall, regime shifts are more likely to share drivers that affect similar ecosystem processes, impact similar ecosystem services, occur in similar ecosystems and occur at similar spatio-temporal scales (S2 Table).

**Fig 2:**
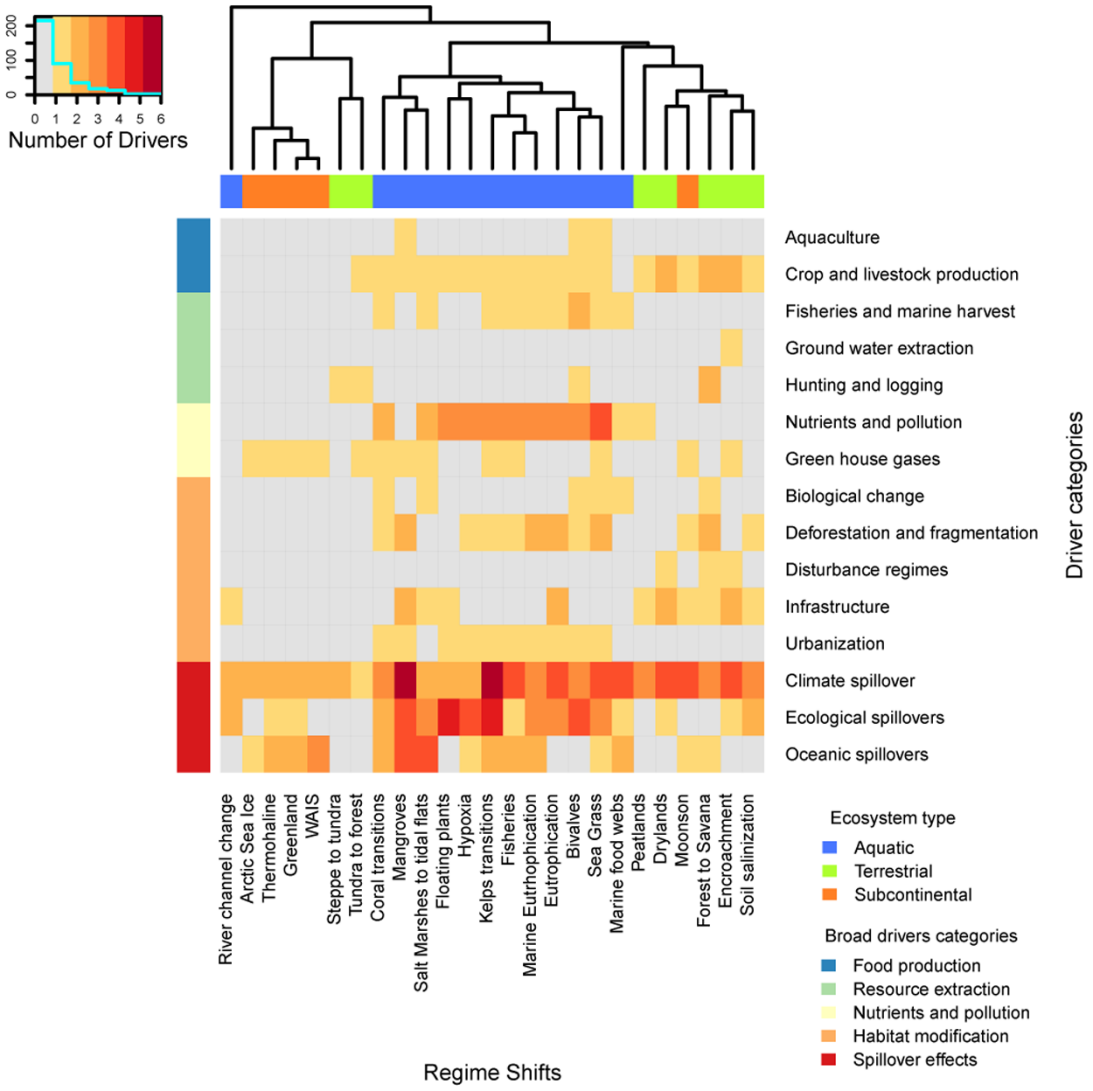
Driver categories per regime shift. Shading intensity indicates the number of drivers per regime shift that falls in each driver category. The dendrogram represents the similarity of regime shifts given the drivers shared (rows) based on hierarchical clustering with an average method upon Jaccard distances. The grey area shows categories with missing drivers. The upper horizontal bar shows the ecosystem type while the left lateral bar shows the 5 broad categories into which the 15 specific drivers categories shown in the rows (right) are classified.

**Fig 3:**
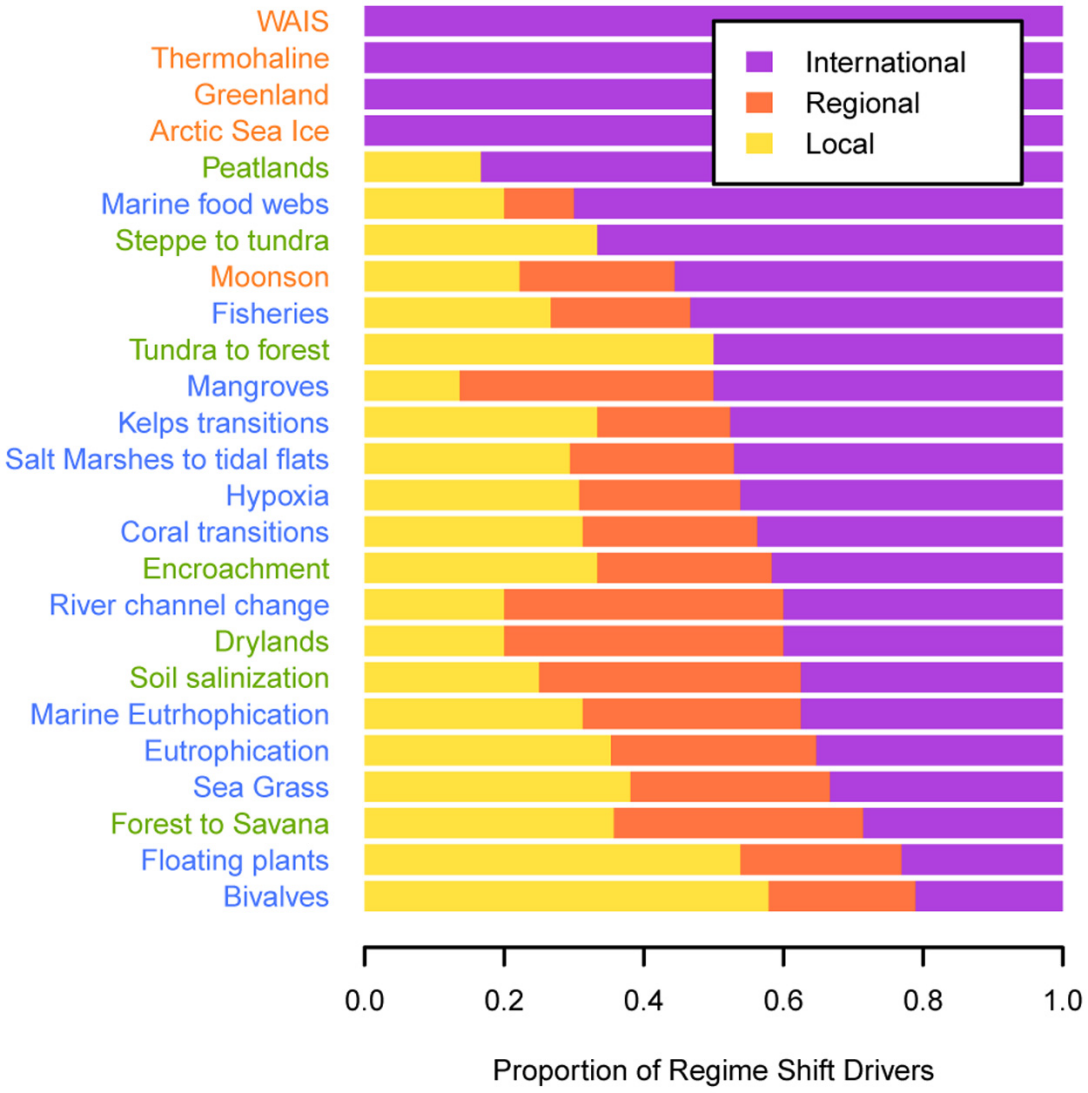
Managerial opportunities per regime shift. Each bar shows the proportion of drivers that can be managed at different scales. Regime shifts names are coloured according to ecosystem type: blue = marine regime shifts, green = terrestrial and orange = subcontinental regime shifts.

Ecosystem type has a strong influence on the variety of regime shift drivers as well as ecosystem services impacted by regime shifts (Figs 2 and 3). Multi-dimensional scaling reveals that aquatic regime shifts often affect fisheries, water purification, disease control and aesthetic values, and they occur more often at the local scale (S2 Fig). Terrestrial regime shifts are strongly influenced by food production and habitat modification, and surprisingly also by climatic spillovers (natural processes that can be amplified or weakened by human action e.g. floods). They consistently affect water cycling, the provision of food crops and fresh water, and occur on land uses related to agriculture. Subcontinental regime shifts are quite different in being almost completely driven by anthropogenic greenhouse gases, climate, ecological, and oceanic spillover effects. Interestingly, they consistently affect climate regulation and occur at time scales of centuries. Based upon our classification of regime shift drivers, we found that climate related drivers are shared across all regime shifts, while oceanic and ecological spillovers are shared across the majority of regime shifts. Aquatic regime shifts are driven by all major types of global change drivers, with no drivers related to terrestrial resource extraction or fire (Fig 2). Almost two thirds of the identified regime shift drivers (62%) have the potential to be managed at local or national scales, while a third (38%) can only be managed internationally (Fig 3).

## Discussion

The variety of drivers revealed by our analysis demonstrates that reducing the risk of regime shifts requires integrated action on multiple dimensions of global change across scales (Figs 2 and 3), a non-trivial challenge for governance. Even heroic actions, such as halting climate change or halting agricultural expansion, if not combined with other actions, will be insufficient to avoid most regime shifts.

Food production and climate change are key drivers of regime shifts that are intertwined with one another (Fig 2) and expected to increase in the coming decades [4,39,40]. These drivers have the potential to synchronize the risk of regime shifts across many systems as well as to produce cascading regime shifts. Cascading effects occur when i) two regime shifts share the same causes increasing their correlation in space or time, ii) when the occurrence of one regime shifts impact the drivers of another increasing the likelihood of a domino effect, and iii) when two regime shifts potentially activate broader feedbacks that interconnect their dynamics, a dynamic also known as cross-scale interactions [41]. Drivers related to food production consist of a broad set of drivers that tend to occur together. They combine resource extraction (e.g. fishing, cropping), nutrients and pollution and strongly co-occur with habitat modification drivers (e.g. urbanization, deforestation), all of which simplify and homogenize ecosystems. Climate related drivers are a more narrow set of connected drivers, providing few opportunities for local or regional management. However in both cases there is strong potential to reduce risk of synchrony by managing local and national scale drivers [42,43]. Local activities and global markets connect climate and food drivers, which increases the risk of synchronized regime shifts, but also provides an opportunity to increase resilience by diversifying local and national energy, food, and regime shift management. For example fishing is a localized activity, but its effects (e.g. collapse of fisheries, marine food webs simplification) and impacts on ecosystem services (e.g. food production) can ripple out globally through the impacts of seafood trade, that increase food demand elsewhere [44].

The number of regime shifts that share climate and food production related drivers furthermore increases the potential for cascading effects among multiple regime shifts. Cascades of regime shifts are possible when some regime shifts enhance the drivers of other types of regime shifts [18,40,45,46]. Regime shifts that contribute to climate change by releasing greenhouse gases or decreasing albedo, or regime shifts that increase the demand for food by e.g. decreasing crop production, can increase the likelihood of other climate or food production driven regime shifts far away. For example, regime shifts involving collapse of the Arctic and Greenland ice sheets would reduce albedo and warm up the climate, increasing the likelihood of fires and warm events that in turn influence regime shifts in the tundra and boreal forest.

It remains unclear whether the observed differences between aquatic, terrestrial and subcontinental regime shifts are explained by the extent to which they have been studied. In the early development of regime shifts theory, aquatic systems were proposed as ideal candidates to test for the existence and mechanisms underlying these non-linear dynamics [19], and consequently have been better studied. Aquatic environments also have and share more drivers, often accounting for land and ocean interactions. Subcontinental regime shifts are harder to study since most evidence relies on observation of long-term processes rather than experimentation. They also share many drivers but to a lesser extent than aquatic regime shifts, and their drivers and impacts are typically climate related. This makes them ideal candidates for the study of cascading effects, when one regime shift acts as a driver of other shifts. Terrestrial regime shifts tend to have more idiosyncratic drivers. They are also prone to cross-scale interactions, when the aggregation of many instances of the same regime shift scales up to affect drivers that further exacerbate the risk of the regime shift elsewhere. Well studied examples of this effect are percolation thresholds for fire, erosion and landscape fragmentation [41,45,47].

Reducing local drivers can build resilience to continued global change, but unless the rates of global change are slowed or reversed, these changes will eventually overwhelm local management [48]. Furthermore, our results (S2 table) suggest that in situations where regime shifts and their drivers are poorly understood, managerial options that work for well-understood regime shifts could potentially be applied to uncertain or data scarce regime shifts if they share similar ecosystem processes, impact similar ecosystem services, occur in similar ecosystems and occur at similar spatio-temporal scales. Similarly, our results suggest that while monitoring direct drivers allows change in the risk of a regime shift to be estimated, management efforts are likely more effective when targeting indirect and generalist drivers (upper part of the bipartite network in Fig 1) because these drivers influence many types of regime shifts, and therefore reducing them can reduce the risk of multiple regime shifts. For example, agriculture, deforestation and erosion are generalist drivers, often operate indirectly, and can be managed at local to regional scales. In combination with monitoring programs checking for changes in other indirect generalist drivers such as sea surface temperature, climate change or nutrient inputs; one can take advantage of windows of opportunity (e.g. wet or dry years related to ENSO) to manage and avoid multiple regime shifts [49].

This paper has presented a novel comparison of regime shifts and their drivers. The development of the regime shift database and the framework for comparison offers a platform for others to extend this work. The regime shifts database framework facilitated comparison of diverse types of regime shifts, broadening our understanding of regime shift similarities at the conceptual level while offering the possibility to translate the observed patterns into useful management insights. Our coding of drivers was done in a systematic, repeatable way, and although some of the categories could have been defined differently, we do not believe it would alter the overall pattern of our results. However, future work needs to take into consideration that the weighting of drivers is not homogeneous across all regime shifts, as such weights are expected to be context dependent. Furthermore, our network approach so far does not allow us to infer the role of dynamics, how changes in the intensity of drivers over time strengthens or weakens their interaction, or how the ordering of events could exacerbate or dampen the effect of such interactions.

Achieving a sustainable future will require meeting needs for ecosystem services [9], while avoiding regime shifts that disrupt the resilient production of these services. Consequently, both theoretical and empirical work is needed to better assess where regime shifts are most likely to happen, which ecosystems and their services will be most affected, and which groups of society will be most impacted. Furthermore, better understanding of the dynamics of regime shifts and their drivers is needed to understand the i) extent to which increasing drivers of global change can trigger synchronous regime shifts; and ii) how regime shifts, by altering the drivers of other regime shifts, can trigger or inhibit cascades of regime shifts.

## Supporting Information

S1 Fig Driver clustering. Shading intensity indicates the similarity between drivers given the regime shifts they cause. The row dendrogram shows a hierarchical clustering calculated on the Sorencen-Dice distance of the drivers matrix. The column side bar shows the scale of management per driver.
(PDF)

S2 Fig Multi-dimensional scaling. Regime shifts are ordered according to the Sorensen-Dice distance given the drivers shared. In panel a) names are coloured according to ecosystem type: blue = marine regime shifts, green = terrestrial and orange = subcontinental regime shifts. Smaller panels show the environmental fitting for subsets of the regime shift categorical variables: b) ecosystem processes (5 variables), c) provisioning services (8), d) regulating services (8), e) cultural services (4), f) drivers (10), g) land use (11), h) scales (8), and i) ecosystem type (11). Only variables that significantly (p<0.05) influence the regime shifts ordering given their shared drivers are shown in purple as vectors, indicating the directionality of their influence.
(PDF)

S1 File A worked regime shift example and causal loop diagram. (PDF)

S1 Table Driver categories. On the left the list of 57 drivers identified which corresponds to the drivers displayed in Fig 1. To facilitate the interpretation of the results we classified drivers into 15 specific categories and 5 broader categories (Fig 2) inspired by existing classification of drivers in references^10,25^. Note that the broad categories are a simple aggregation of the specific ones given that specific categories are mutually exclusive.
(DOCX)

S2 Table Summary of exponential random graph models fitted to the bipartite network data. Models 01 to 05 are null models following the specifications for bipartite networks^31–34^. Model 01 is a Markov random model. Model 02 explores the effect of 2 and 3 paths on both projections of the bipartite network (terms b1star2, b1star3, b2star2 and b2star3). Model 03 explore the effects of three-paths and cycles also known as clustering model. Model 04 is a curved exponential model that show the effects of geometrically weighted node shared partners (GWNSP), this is the number of open triangles that simultaneously share two basal nodes, thus a proxy for drivers or regime shifts co-occurrence. The weight of such number is adjusted with parameter alpha. Model 05 adds geometrically weighted terms for the degree (number of links) on each one-mode projection. Model 41 is the model that exhibited the best fit following both Akaike Information Criterion (AIC) and Maximum Likelihood Estimation (MLE). Model 41 combines a curved exponential model and explores the effects of homophily—the likelihood of two nodes of being connected on the one-mode projections given that they share attributes: scale of management for driver nodes, ecosystem type of regime shifts nodes, and nestedness and frequency as node covariates respectively. All model are dyadic dependent, only model 41 do not exhibit degeneracy. Significance levels: ***P<0.001, **P<0.01, *P<0.05, ·P< 0.1
(DOCX)

S3 Table Summary of exponential random graph models fitted to one-mode network data. Models fitted for the one-mode projections have weighted links, therefore null models only count non-zero links and its sum^37^, taking as reference mode the Poisson distribution (Both Mod.RS.Null and Mod.D.Null). Two models were fitted on the regime shifts projection: Mod.RS.1 tested the effect of homophily (‘Nodematch’) on ecosystem type, this is whether the likelihood of two regime shifts sharing drivers is influenced by occurring on the same ecosystem type. The term ‘Nodefactor’ tested whether the likelihood is influenced by each of the ecosystem types taken as a factor for the regime shifts network. Node covariates was tested for nestedness, number of papers on the ISI web of science, and frequency. On the one-mode projections, frequency is measured as the number of links on the bipartite network over all possible number of links. The second model on the regime shifts network (Mod.RS.2) complemented the first by adding an extra set of terms that assessed the edge covariates with the information from the regime shifts database (RSDB). For the drivers network projection (Mod.D.1) homophily was assessed for a match on the driver’s scale of management and match on the driver’s categories (Fig 2). The effect of each variable as factors was assessed for the scale of management, and node covariates were tested for nestedness, directedness and frequency. The best models fitted were Mod.RS.2 for regime shifts and Mod.D.1 for drivers following both Akaike Information Criterion (AIC) and Maximum Likelihood Estimation (MLE). All model are dyadic dependent, and none of them exhibit degeneracy. Significance levels: ***P<0.001, **P<0.01, *P<0.05, ·P< 0.1
(DOCX)

## References

[1] Barnosky AD, Hadly EA, Bascompte J, Berlow EL, Brown JH, Fortelius M, et al Approaching a state shift in Earth’s biosphere. Nature. Nature Publishing Group; 2012;486: 52–58. 10.1038/nature11018 22678279

[2] Rockström J, Steffen W, Noone K, Persson Å, Chapin F, Lambin E, et al A safe operating space for humanity. Nature. 2009;461: 472–475. 10.1038/461472a 19779433

[3] Lenton T, Held H, Kriegler E, Hall J, Lucht W, Rahmstorf S, et al Tipping elements in the Earth's climate system. P Natl Acad Sci Usa. 2008;105: 1786. 10.1073/pnas.0705414105 PMC2538841 18258748

[4] Steffen W, Crutzen PJ, McNeill JR. The Anthropocene: Are humans now overwhelming the great forces of nature. Ambio. 2007;36: 614–621. 18240674 10.1579/0044-7447(2007)36[614:taahno]2.0.co;2

[5] Steffen W, Broadgate W, Deutsch L, Gaffney O, Ludwig C. The trajectory of the Anthropocene: The Great Acceleration The Anthropocene Review. SAGE Publications; 2015;: 2053019614564785 10.1177/2053019614564785

[6] Millennium Ecosystem Assessment Ecosystems and human well-being: synthesis‎. Washington, DC.: Island Press; 2005.

[7] Scheffer M, Carpenter S. Catastrophic regime shifts in ecosystems: linking theory to observation. Trends Ecol Evol. 2003;18: 648–656. 10.1016/j.tree.2003.09.002

[8] Scheffer M, Carpenter S, Foley J, Folke C, Walker B. Catastrophic shifts in ecosystems. Nature. 2001;413: 591–596. 11595939 10.1038/35098000

[9] Carpenter SR, Mooney HA, Agard J, Capistrano D, DeFries RS, Díaz S, et al Science for managing ecosystem services: Beyond the Millennium Ecosystem Assessment. P Natl Acad Sci Usa. 2009;106: 1305–1312. 10.1073/pnas.0808772106 PMC2635788 19179280

[10] Folke C, Carpenter S, Walker B, Scheffer M, Elmqvist T, Gunderson L, et al Regime shifts, resilience, and biodiversity in ecosystem management. Annu Rev Ecol Evol S. 2004;35: 557–581. 10.1146/annurev.ecolsys.35.021103.105711

[11] Gordon LJ, Peterson GD, Bennett EM. Agricultural modifications of hydrological flows create ecological surprises. Trends Ecol Evol. 2008;23: 211–219. 10.1016/j.tree.2007.11.011 18308425

[12] Scheffer M, Carpenter SR, Lenton TM, Bascompte J, Brock W, Dakos V, et al Anticipating Critical Transitions. Science. 2012;338: 344–348. 10.1126/science.1225244 23087241

[13] Karlsson JM, Bring A, Peterson GD, Gordon LJ, Destouni G. Opportunities and limitations to detect climate-related regime shifts in inland Arctic ecosystems through eco-hydrological monitoring EPL (Europhysics Letters). IOP Publishing; 2011;6: 014015 10.1088/1748-9326/6/1/014015

[14] Rocha J, Yletyinen J, Biggs R, Blenckner T, Peterson G. Marine regime shifts: drivers and impacts on ecosystems services. Phil Trans R Soc B. 2015;: 20130273 10.1098/rstb.2013.0273

[15] Ban SS, Graham NAJ, Connolly SR. Evidence for multiple stressor interactions and effects on coral reefs. Glob Change Biol. 2014;20: 681–697. 10.1111/gcb.12453 24166756

[16] Geist H, Lambin E. Dynamic causal patterns of desertification. BioScience. 2004;54: 817–829.

[17] Halpern BS, Walbridge S, Selkoe KA, Kappel CV, Micheli F, D'Agrosa C, et al A global map of human impact on marine ecosystems. Science. 2008;319: 948–952. 10.1126/science.1149345 18276889

[18] Hughes TP, Carpenter S, Rockström J, Scheffer M, Walker B. Multiscale regime shifts and planetary boundaries. Trends Ecol Evol. 2013;28: 389–395. 10.1016/j.tree.2013.05.019 23769417

[19] Holling CS. Resilience and stability of ecological systems. Annual review of ecology and systematics. 1973;4: 1–23.

[20] Noy-Meir I. Stability of grazing systems: an application of predator-prey graphs. The Journal of Ecology. 1975.

[21] Carpenter SR. Regime shifts in lake ecosystems. Ecology Institute; 2003.

[22] Scheffer M, Bascompte J, Brock WA, Brovkin V, Carpenter SR, Dakos V, et al Early-warning signals for critical transitions. Nature. 2009;461: 53–59. 10.1038/nature08227 19727193

[23] Biggs RO, Peterson GD, Rocha JC. The Regime Shifts Database: A framework for analyzing regime shifts in social-ecological systems. bioRxiv. Cold Spring Harbor Labs Journals; 2015;: 018473 10.1101/018473

[24] Greene CH, Pershing AJ, Cronin TM, Ceci N. Arctic Climate Change and its Impacts on the Ecology of the North Atlantic. Ecology. 2008;89: S24–S38. 19097482 10.1890/07-0550.1

[25] Nelson GC, Bennett E, Berhe AA, Cassman KG, DeFries R, Dietz T, et al Anthropogenic drivers of ecosystem change: an overview. Ecol Soc. 2006;11 Available: http://www.ecologyandsociety.org/vol11/iss2/art29/

[26] Lane D. The emergence and use of diagramming in system dynamics: a critical account. Systems Research and Behavioral Science. 2008;25: 3–23.

[27] Newman M, Strogatz S, Watts D. Random graphs with arbitrary degree distributions and their applications. Phys Rev E. 2001;64. 10.1103/PhysRevE.64.026118 11497662

[28] Roberts A, Stone L. Island-sharing by archipelago species. Oecologia. 1990;83: 560–567. 10.1007/BF00317210 28313193

[29] R Core Team. R: A Language and Environment for Statistical Computing [Internet]. Vienna, Austria: R Foundation for Statistical Computing; 2012 Jan. Available: http://www.R-project.org/

[30] Admiraal R, Handcock MS. Networksis: a package to simulate bipartite graphs with fixed marginals through sequential importance sampling. J Stat Softw. 2008;24: 1–21. 29129971 10.18637/jss.v024.i08 PMC5679483

[31] Hunter DR, Handcock MS, Butts CT, Goodreau SM, Morris M. ergm: A Package to Fit, Simulate and Diagnose Exponential-Family Models for Networks. J Stat Softw. 2008;24: nihpa54860 19756229 10.18637/jss.v024.i03 PMC2743438

[32] Robins G, Morris M. Advances in exponential random graph (p*) models. Social networks. 2007;29: 169–172. 10.1016/j.socnet.2006.08.004 PMC2031833 18449326

[33] Fortuna MA, Stouffer DB, Olesen JM, Jordano P, Mouillot D, Krasnov BR, et al Nestedness versus modularity in ecological networks: two sides of the same coin? J Anim Ecol. 2010 10.1111/j.1365-2656.2010.01688.x 20374411

[34] Wang P, Pattison P, Robins G. Exponential random graph model specifications for bipartite networks—A dependence hierarchy. Social networks. 2013;35: 211–222. 10.1016/j.socnet.2011.12.004

[35] Hunter DR. Curved exponential family models for social networks. Social networks. 2007;29: 216–230. 10.1016/j.socnet.2006.08.005 18311321 PMC2031865

[36] Snijders TAB. Markov chain Monte Carlo estimation of exponential random graph models. Journal of Social Structure. Citeseer; 2002;3: 1–40.

[37] Krivitsky PN. Exponential-family random graph models for valued networks. Electronic Journal of Statistics. Institute of Mathematical Statistics; 2012;6: 1100–1128. 10.1214/12-EJS696 PMC3964598 24678374

[38] Warnes GR, Ben Bolker IRSCADCB, Bonebakker L, Gentleman R, Liaw WHA, Lumley T, et al gplots: Various R programming tools for plotting data [Internet]. 2nd ed Available: http://CRAN.R-project.org/package=gplots

[39] Foley JA, Ramankutty N, Brauman KA, Cassidy ES, Gerber JS, Johnston M, et al Solutions for a cultivated planet. Nature. Nature Publishing Group; 2011;478: 337–342. 10.1038/nature10452 21993620

[40] Lenton TM, Williams HTP. On the origin of planetary-scale tipping points. Trends Ecol Evol. 2013;28: 380–382. 10.1016/j.tree.2013.06.001 23777818

[41] Peters Debra P. C., Pielke Roger A S, Bestelmeyer BT, Allen CD, Munson-McGee S, Havstad KM. Cross-scale interactions, nonlinearities, and forecasting catastrophic events. P Natl Acad Sci Usa. National Acad Sciences; 2004;101: 15130–15135. 10.1073/pnas.0403822101 PMC523446 15469919

[42] Kennedy EV, Perry CT, Halloran PR, Iglesias-Prieto R, Schönberg CHL, Wisshak M, et al Avoiding Coral Reef Functional Collapse Requires Local and Global Action. Current Biology. Elsevier; 2013 10.1016/j.cub.2013.04.020 23664976

[43] Brook BW, Ellis EC, Perring MP, Mackay AW, Blomqvist L. Does the terrestrial biosphere have planetary tipping points? Trends Ecol Evol. 2013;28: 396–401. 10.1016/j.tree.2013.01.016 23453050

[44] Crona BI, Daw TM, Swartz W, Norström AV, Nyström M, Thyresson M, et al Masked, diluted and drowned out: how global seafood trade weakens signals from marine ecosystems. Fish Fish. 2015;: n/a–n/a. 10.1111/faf.12109

[45] Peters DPC, Sala OE, Allen CD, Covich A, Brunson M. Cascading events in linked ecological and socioeconomic systems. FRONTIERS IN ECOLOGY. 2007;5: 221–224.

[46] Kinzig A, Ryan P, Etienne M, Allison H, Elmqvist T, Walker B. Resilience and regime shifts: assessing cascading effects. Ecol Soc. 2006;11: 20.

[47] Abades SR, Gaxiola A, Marquet PA. Fire, percolation thresholds and the savanna forest transition: a neutral model approach. J Ecology. 2014;102: 1386–1393. 10.1111/1365-2745.12321

[48] Scheffer M, Barrett S, Carpenter SR, Folke C, Green AJ, Holmgren M, et al Creating a safe operating space for iconic ecosystems. Science. American Association for the Advancement of Science; 2015;347: 1317–1319. 10.1126/science.aaa3769 25792318

[49] Holmgren M, Scheffer M. El Nino as a window of opportunity for the restoration of degraded arid ecosystems. Ecosystems. 2001;4: 151–159.

[50] Estes JA, Terborgh J, Brashares JS, Power ME, Berger J, Bond WJ, et al Trophic downgrading of planet Earth. Science. American Association for the Advancement of Science; 2011;333: 301–306. 10.1126/science.1205106 21764740

[51] Diaz RJ, Rosenberg R. Spreading Dead Zones and Consequences for Marine Ecosystems. Science. 2008;321: 926–929. 10.1126/science.1156401 18703733

[52] Jackson J, Kirby M, Berger W, Bjorndal K, Botsford L, Bourque B, et al Historical Overfishing and the Recent Collapse of Coastal Ecosystems. Science. 2001;293: 629–637. 10.1126/science.1059199 11474098

[53] Scheffer M, Szabo S, Gragnani A, van Nes E, Rinaldi S, Kautsky N, et al Floating plant dominance as a stable state. P Natl Acad Sci Usa. 2003;100: 4040–4045. 10.1073/pnas.0737918100 PMC153044 12634429

[54] Dent CL, Cumming GS, Carpenter SR. Multiple states in river and lake ecosystems. Philos Trans R Soc Lond, B, Biol Sci. 2002;357: 635–645. 10.1098/rstb.2001.0991 12079525 PMC1692979

[55] Duke NC, Meynecke JO, Dittmann S, Ellison AM. A world without mangroves? Science. 2007. 10.1126/science.317.5834.41b 17615322

[56] Hughes BB, Eby R, Van Dyke E, Tinker MT, Marks CI, Johnson KS, et al Recovery of a top predator mediates negative eutrophic effects on seagrass. P Natl Acad Sci Usa. National Acad Sciences; 2013;110: 15313–15318. 10.1073/pnas.1302805110 PMC3780875 23983266

[57] Rabalais NN, Turner RE, Díaz RJ, Justić D. Global change and eutrophication of coastal waters. Oxford University Press; 2009;66: 1528–1537. 10.1093/icesjms/fsp047

[58] Paolo FS, Fricker HA, Padman L. Volume loss from Antarctic ice shelves is accelerating. Science. American Association for the Advancement of Science; 2015;: aaa0940 10.1126/science.aaa0940 25814064

[59] Powell EN, Ashton-Alcox KA, Kraeuter JN, Ford SE, Bushek D. Long-term trends in oyster population dynamics in Delaware Bay: regime shifts and response to disease. J Shellfish Res. BioOne; 2008;27: 729–755.

[60] Bellwood D, Hughes T, Folke C, Nyström M. Confronting the coral reef crisis. Nature. 2004;429: 827–833. 15215854 10.1038/nature02691

[61] Ling S, Johnson C, Frusher S, Ridgway K. Overfishing reduces resilience of kelp beds to climate-driven catastrophic phase shift. P Natl Acad Sci Usa. 2009;106: 22341–22345. 10.1073/pnas.0907529106 PMC2793314 20018706

[62] Roques K, O'Connor T, Watkinson A. Dynamics of shrub encroachment in an African savanna: relative influences of fire, herbivory, rainfall and density dependence. J Appl Ecol. 2001;38: 268–280.

[63] Rozema J, Flowers T. Ecology. Crops for a salinized world. Science. American Association for the Advancement of Science; 2008;322: 1478–1480. 10.1126/science.1168572 19056965

[64] Hirota M, Holmgren M, van Nes EH, Scheffer M. Global resilience of tropical forest and savanna to critical transitions. Science. American Association for the Advancement of Science; 2011;334: 232–235. 10.1126/science.1210657 21998390

[65] Bonan GB. Forests and climate change: forcings, feedbacks, and the climate benefits of forests. Science. American Association for the Advancement of Science; 2008;320: 1444–1449. 10.1126/science.1155121 18556546

[66] Sahana AS, Ghosh S, Ganguly A, Murtugudde R. Shift in Indian summer monsoon onset during 1976/1977 Environmental Research Letters. IOP Publishing; 2015;10: 054006 10.1088/1748-9326/10/5/054006

[67] Turetsky MR, Benscoter B, Page S, Rein G, van der Werf GR, Watts A. Global vulnerability of peatlands to fire and carbon loss. Nature Geoscience. Nature Publishing Group; 2015;8: 11–14. 10.1038/ngeo2325

[68] Gregory JM, Huybrechts P, Raper SCB. Climatology: threatened loss of the Greenland ice-sheet. Nature. 2004;428: 616 10.1038/428616a 15071587

[69] Stouffer RJ, Yin J, Gregory JM, Dixon KW, Spelman MJ, Hurlin W, et al Investigating the causes of the response of the thermohaline circulation to past and future climate changes. J Climate. 2006;19: 1365–1387.

[70] Koppel JV de, Wal DVD, Bakker JP, Herman PMJ. Self-Organization and Vegetation Collapse in Salt Marsh Ecosystems. Am Nat. 2005;165: E1–E12. 10.1086/426602 15729634

[71] Livina VN, Lenton TM. A recent tipping point in the Arctic sea-ice cover: abrupt and persistent increase in the seasonal cycle since 2007. The Cryosphere. 2013;7: 275–286. 10.5194/tc-7-275-2013

[72] Zimov SA, Chuprynin VI, Oreshko AP, Chapin FS III. Steppe-tundra transition: a herbivore-driven biome shift at the end of the Pleistocene. Am Nat. 1995.

