# Supplementary figures and images for: Regime Shifts in the Anthropocene: Drivers, Risks, and Resilience
Source: PLoS One. 2015 Aug 12;10(8):e0134639. doi: 10.1371/journal.pone.0134639 (PMC4533971; doi:10.1371/journal.pone.0134639)

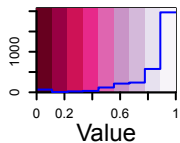

# Management scale

- International
- Regional
- Local

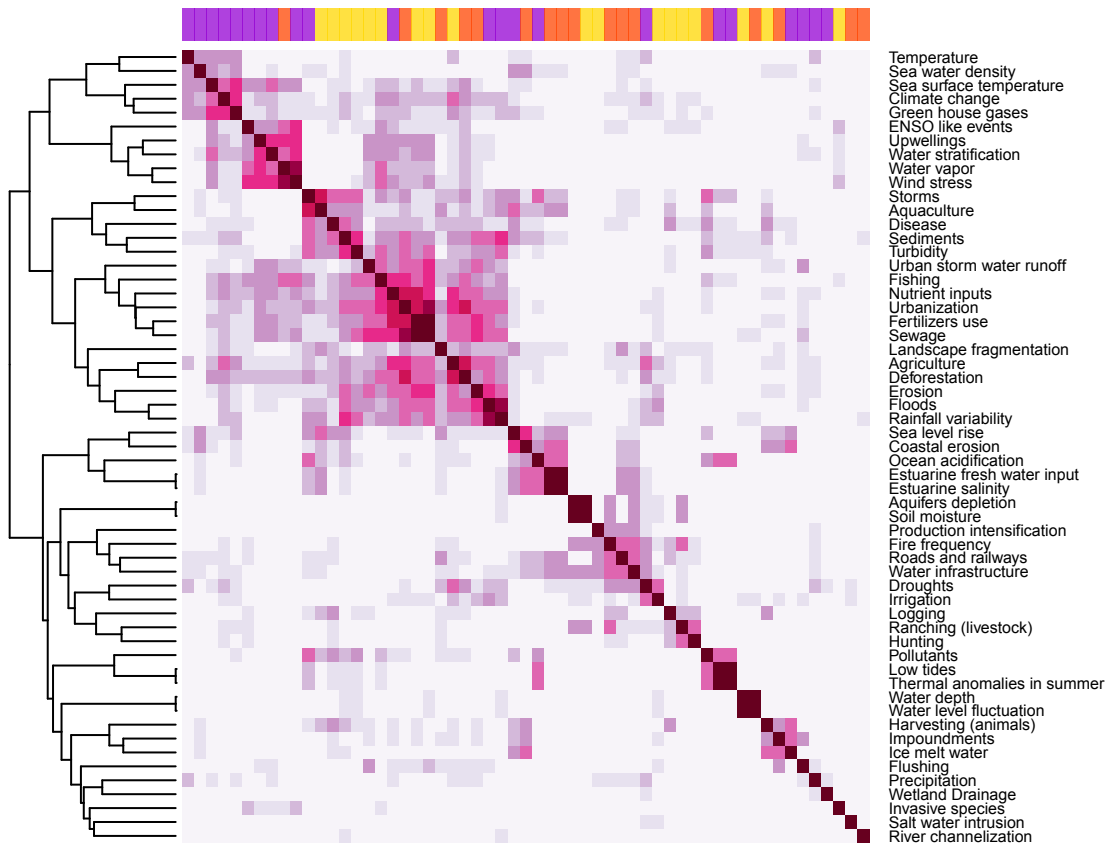

Supplement: S1 Fig — Shading intensity indicates the similarity between drivers given the regime shifts they cause. The row dendrogram shows a hierarchical clustering calculated on the Sorencen-Dice distance of the drivers matrix. The column side bar shows the scale of management per driver. (PDF) [file pone.0134639.s001.pdf]
