# Supplementary material for: Regime Shifts in the Anthropocene: Drivers, Risks, and Resilience
Source: PLoS One. 2015 Aug 12;10(8):e0134639. doi: 10.1371/journal.pone.0134639 (PMC4533971; doi:10.1371/journal.pone.0134639)
Supplement: S2 Fig — Regime shifts are ordered according to the Sorensen-Dice distance given the drivers shared. In panel a) names are coloured according to ecosystem type: blue = marine regime shifts, green = terrestrial and orange = subcontinental regime shifts. Smaller panels show the environmental fitting for subsets of the regime shift categorical variables: b) ecosystem processes (5 variables), c) provisioning services (8), d) regulating services (8), e) cultural services (4), f) drivers (10), g) land use (11), h) scales (8), and i) ecosystem type (11). Only variables that significantly (p<0.05) influence the regime shifts ordering given their shared drivers are shown in purple as vectors, indicating the directionality of their influence. (PDF) [file pone.0134639.s002.pdf]

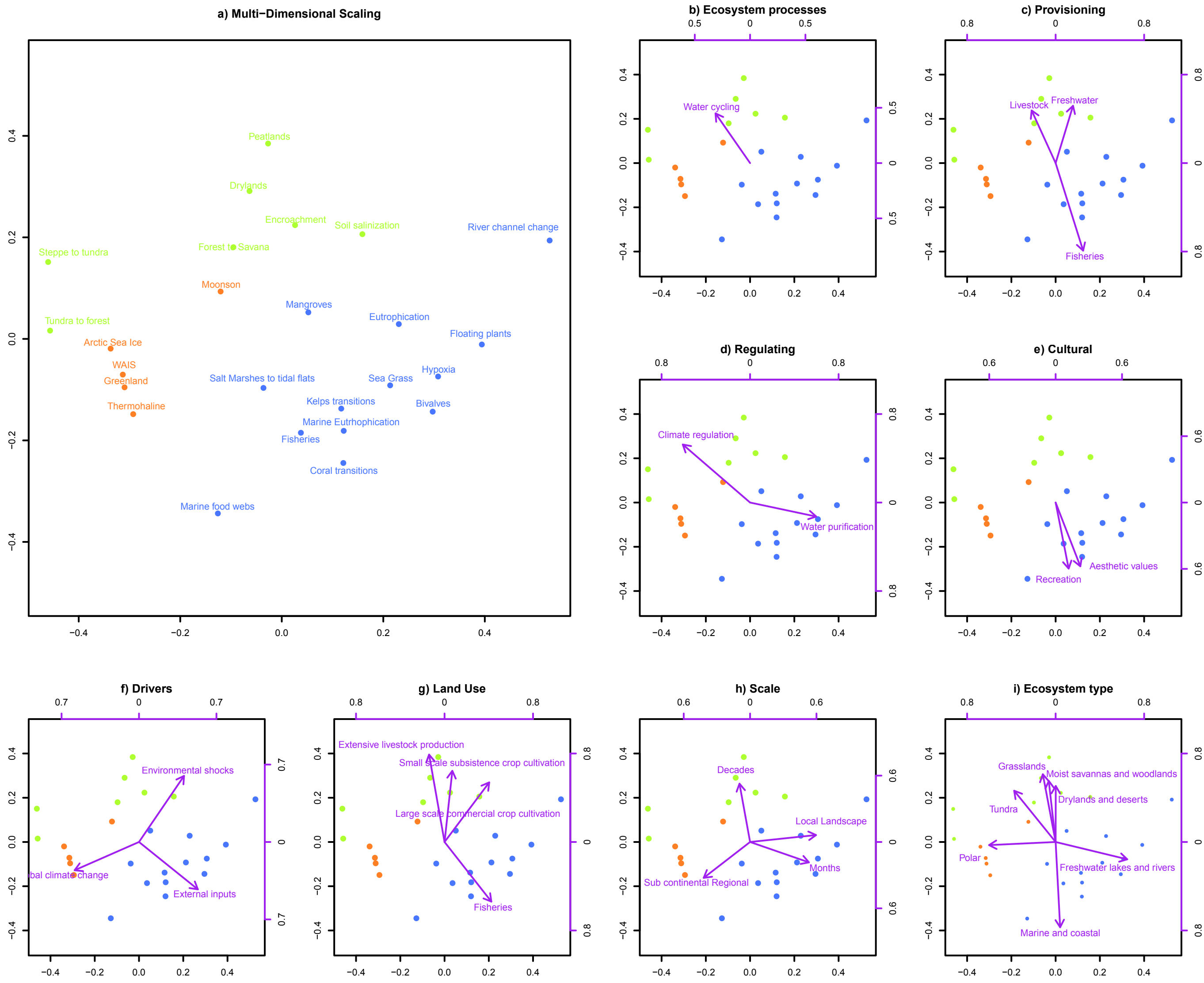

a) Multi−Dimensional Scaling
Peatlands
Drylands
Encroachment
Soil salinization
River channel change
Steppe to tundra
Forest to Savana
Moonson
Mangroves
Eutrophication
Tundra to forest
Floating plants
Arctic Sea Ice
WAIS
Greenland
Salt Marshes to tidal flats
Sea Grass
Hypoxia
Thermohaline
Kelps transitions
Bivalves
Marine Eutrhophication
Fisheries
Coral transitions
Marine food webs
b) Ecosystem processes
Water cycling
c) Provisioning
Livestock
Freshwater
Fisheries
d) Regulating
Climate regulation
Water purification
e) Cultural
Aesthetic values
Recreation
f) Drivers
Environmental shocks
bal climate change
External inputs
g) Land Use
Extensive livestock production
Small scale subsistence crop cultivation
Large scale commercial crop cultivation
Fisheries
h) Scale
Decades
Local Landscape
Months
Sub continental Regional
i) Ecosystem type
Grasslands
Moist savannas and woodlands
Tundra
Drylands and deserts
Polar
Freshwater lakes and rivers
Marine and coastal
