# Supplementary material for: Regime Shifts in the Anthropocene: Drivers, Risks, and Resilience
Source: PLoS One. 2015 Aug 12;10(8):e0134639. doi: 10.1371/journal.pone.0134639 (PMC4533971; doi:10.1371/journal.pone.0134639)
Supplement: S1 File — (PDF) [file pone.0134639.s003.pdf]

Supplementary Information for

Regime Shifts in the Anthropocene: drivers, risk, and resilience

by Juan-Carlos Rocha, Garry D. Peterson & Reinette O. Biggs.

This document presents a worked example of a regime shift. We first provide a synthesis of the regime shift dynamics and a causal loop diagram (CLD). We then compare the resulting CLD with other regime shifts to make explicit the role of direct and indirect drivers. For each regime shift analyses in this study we did a similar literature synthesis and CLD which are available in the regime shift database. A longer version of this working example and CLDs can be found at www.regimeshifts.org.

1. Kelps transitions

Summary. Kelp forests are marine coastal ecosystems located in shallow areas where large macroalgae ecologically engineer the environment to produce a coastal marine environmental substantially different from the same area without kelp. Kelp forests can undergo a regime shift to turf-forming algae or urchin barrens. These shift leads to loss of habitat and ecological complexity. Shifts to turf algae are related to nutrient input, while shifts to urchin barrens are related to trophic-level changes. The consequent loss of habitat complexity may affect commercially important fisheries. Managerial options include restoring biodiversity and installing wastewater treatment plants in coastal zones.

Kelps are marine coastal ecosystems dominated by macroalgae typically found in temperate areas. This group of species form submarine forests with three or four layers, which provides different habitats to a variety of species. These forests maintain important industries like lobster and rockfish fisheries, the chemical industry with products to be used in e.g. toothpaste; and the tourism industry of recreational diving and kayaking. Shifts from kelp canopies to other regimes like urchin barrens and turfed landscapes has been reported all over the world[1-4]. Kelp forest dynamics usually include cycles of disturbance-recovery. However, increasing human-dominated coastlines modify environmental conditions that facilitate other regimes like turfs or urchin barrens, which in turn inhibit the reestablishment of kelp forest.

Alternative regimes

This regime shift is associated with a change in habitat in shallow marine coastal ecosystems. Three different self-reinforcing regimes can be identified:

Kelp forests are highly productive ecosystems dominated by canopy-formed algae in cold-water rocky marine coastlines. Among the biota associated with kelp forest are marine mammals, fishes, crabs, sea urchins, mollusks, other algae and epibiota [1]. At least four trophic levels are found in kelp forests, and apex predators such as sea otters, cod, pollock, hake, and haddock are common. These predators control lower

trophic levels, and especially sea urchins, the main herbivore, are kept in low numbers. In addition, urchins are prevented from grazing in the kelp forest through the effect of kelp foliage sweeping over the rocks due to its flexibility and the force of waves [5].

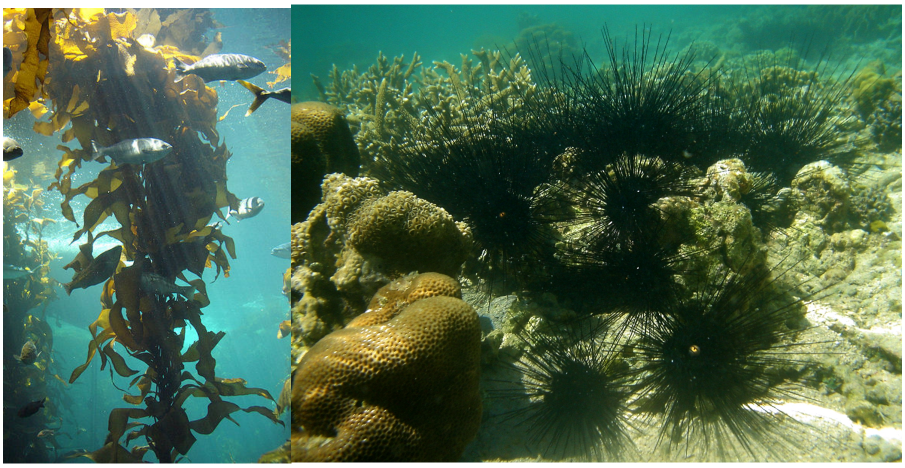

Figure 1. Kelp forest on left and urchin barren on the right. Both pictures are under creative commons available at www.commons.wikimedia.org

Urchin barrens are formed where the kelp canopy has been deforested by urchins. Trophic-level cascades are responsible for urchin overabundance. Overfishing of the apex predators can lead to shifts in the dominance of consumers at lower trophic levels[6]. This has been particularly observed in the case of overfishing of sea otter, cod and haddock, which have released urchins from their predatory controls. Large populations of urchins form grazing fronts that graze down strongly on kelp forest and can survive in adverse conditions by feeding on turf-forming algae [7].

In some cases trophic-level cascades have allowed other species to reach sufficient population sizes to exert some control on urchins, for instance lobsters and crabs in the North Atlantic[1]. In addition, declines in predatory fish have created a market for urchins, especially in Japan, establishing a new human induced control on the herbivore. In some cases, harvesting of urchins for this market has led to the re-establishment of kelp forest, despite the urchin fishery being prohibited [1,8].

Turf-forming algae may be considered another regime where opportunistic species with simple and less diverse elements dominate a seascape previously dominated by kelp, a perennial species with structurally complex community [9]. The feedback is given by the ability of turf-forming algae to persist under conditions of elevated nutrients, frequently attributed to coastal urban settlements, inhibiting the recruitment of kelp species [2].

Once the system shifts from one regime to another, the main ecosystem impact is the loss of habitat complexity due to kelp defoliation. Kelp is a three dimensional structure that offers shelter and food for many species; urchin barrens and turfs do not have this characteristic. This loss is associated with reduction of food web complexity and loss of functional groups[6], with varying effects on fisheries. Some valuable fish species may diminish since kelp forests provide nursery areas. However, invertebrate species such as lobster and crab have shown increases in population[1]. The abundance of such lower-level consumers reflects an exacerbation of the “fishing down food

web" effect[10]. The ecosystem service impacts of algae turfs are likely to be similar to those related to coastal eutrophication. Such effects include abundance of rich-nutrient environment species as shellfish, bad odors and the associated consequences for recreational and aesthetic values. In addition, kelps support a multimillion dollar industry of canopy-cropping for alginates[1]. This product is commercially important in pharmaceutical and chemical industry.

Feedback mechanisms

The kelp regime is maintained by a healthy food web, usually with four trophic levels that keep urchin populations under control. The urchin regime is maintained by reduction of predators due to lack of habitat or fishing pressure. The turf-forming algae regime is maintained by an environment over-enriched by nutrients either from sediments from land or upwelling nutrients from the deep ocean. Note that canopy-forming algae and turf-forming algae are functional groups competing for resources and space in the ecosystem. All feedbacks are local and well established.

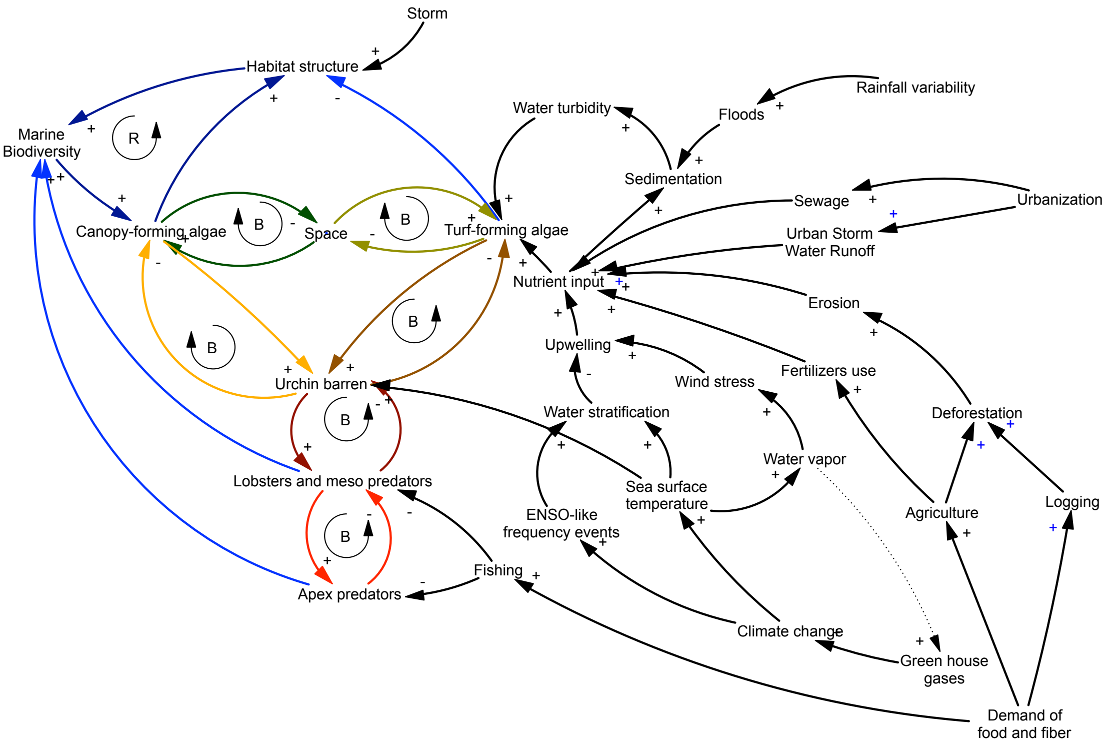

Figure 2. Causal loop diagram for Kelp Transitions. Different feedbacks are colored and marked R for reinforcing feedbacks and B for balancing ones. Variables that are outside the feedback mechanism are drivers since their dynamics are not determined by the feedbacks in the system. Driver links are depicted black. Developed by Juan Rocha (2010, source: www.regimeshifts.org)

Kelp Forest

- ***Competition feedback (Local, well established)***: Canopy forming algae and turf forming algae are two set of species that compete for space, light and nutrients. The consumption of such resources reduces the availability of the resource for its own progeny and for other species. For this reason, this relationship is represented as a balancing feedback loop. There are two competition feedbacks in figure 2 (light and dark green). The resulting

ecosystem is a kelp forest when canopy-forming algae is dominant, reducing the possibility of turf-forming algae to develop.

- ***Predation feedback (Local, well established)***: The relationship between predator and prey in the food web can be viewed as a feedback. In a nutshell, the more abundant the prey is, the more resource the predator has and vice-versa, producing a balancing feedback loop (yellow, brown and red in Fig 2). This feedback is represented in the CLD aggregated across functional groups: between apex predators and lobsters - meso-predators; between the latter and urchins; and between urchins and algae groups. Lobsters and other meso-predators help maintain kelp forests by regulating urchin populations at low densities.
- ***Structure feedback (Local, well established)***: Canopy forming macroalgae maintain more complex habitat structure that in turns favor the presence of high biodiversity (dark and light blue feedbacks in Fig 2). When diverse predatory species are present in the ecosystem, these predators regulate urchin populations, which maintains kelp forests.

Urchin barren

- ***Predation feedback (Local, well established)***: The ecosystem is dominated by urchin barrens when the predation feedback amongst urchins and kelps is strong, while the predation feedback amongst lobster or meso-predators and urchins is weak. It may be related to changes in water temperature that favors urchin barren establishment, or fishing pressure that reduces meso-predator abundance.
- ***Structure feedback (Local, well established):*** As urchin barrens dominate, less structural habitat complexity is provided by kelp forest. Thus, meso-predators habitat requirements may be affected, reducing their abundance and the predation pressure on urchins.

Turf-forming algae

- ***Competition feedback (Local, well established):*** In turf-forming algae dominance regime, the competition feedback is favoring turfs, reducing space, nutrients and light availability for kelps to develop.

Drivers

Two key direct drivers are identified: overfishing functional groups[1,6] and input of nutrients[2-4]. The latter is related to deposition of wastewater from urban settlements and agriculture in adjacent catchments. Strong rain events and floods represent shock events for kelp ecosystems given the pulse input of nutrients. El Niño or global warming events may generate water stratification. As consequence, nitrogen concentration declines and kelps become nitrogen limited[1]. In addition pollution discharges and sedimentation may play a synergistic role as stressors. In Tasmania for example, global warming has favored the reproduction of urchins which in synergy with lobster fishing has reduced kelp resilience[3,4].

Gorman and Connell (2009) also report that the loss of kelp dominated areas undermine kelp's ability to reestablish in disturbed areas. While deforested areas surrounded by kelp patches are more likely to return to the kelp regime, isolated kelp disturbed patches are more likely to stick in the turfed regime.

Important shocks (e.g. droughts, floods) that contribute to the regime shift include:

- *Rain and floods (regional, well established)*: Strong rain events and floods represent shock events for kelp ecosystems given the pulse input of nutrients or by perturbing habitat structure. Nutrients in turn unbalance the competition between kelps and turfs favoring the development of the latter; which can use the excess of nutrients faster than kelps and also take advantage of the turbidity conditions generated by nutrients.
- *ENSO (global, well established)*: El Niño events or global warming events may generate water stratification. As a consequence, nitrogen concentration declines and kelps become nitrogen limited[1]. In addition pollution discharges and sedimentation may play a synergetic role as stressors.

The main external direct drivers that contribute to the shift include:

- *Overfishing (regional, well established):* overfishing functional groups is one of the most important drivers of kelp transitions[1,3,6,11]. Fishing pressure reduces control of mid predators on urchins favoring the formation of turfs. When fishing is strong enough on urchins, it may favor the formation of turfs as well.
- *Nutrients inputs (regional, well established)*: Input of nutrients is another key driver of the regime shift, both natural from deep ocean upwelling or anthropogenic runoff [2]. Nutrients inputs increase sedimentation and turbidity, favoring conditions for turf to outcompete kelps.

The main external indirect drivers that contribute to the shift are:

- *Food demand (local-regional, speculative):* Higher food demand usually stimulates agriculture, both as expansion of agricultural frontier or increase of fertilizers use to increase yield. It also increases fishing pressure on the food web.
- *Agriculture (regional, well established):* Agriculture often requires the use of fertilizers. When soils are eroded or washed, fertilizers run downstream increasing nutrient input to lakes and rivers.
- *Urban growth (global, well established):* Urban growth in coastal zones increases the production of sewage that is rich in nutrients. It also increases the water runoff on the urban landscape, which transports nutrients into coastal water.
- *Deforestation (regional, well established):* Deforestation and poor agricultural management can accelerate, in magnitude and frequency, the nutrient runoff from agricultural lands. Deforestation increases landscape fragmentation and facilitates landscape conversion to agriculture. Both reduce the capacity of the landscape to retain water in the soil, accelerating erosive processes and runoff of nutrients [12].
- *Global warming (regional, speculative):* Global warming is expected to increase average water surface temperature. It is also expected to increase the gradient between land and ocean temperatures, strengthening winds parallel to the coast and as result increasing upwelling of deep ocean water[13]. This could increase nutrient inputs to coastal ecosystems at the regional scale. On the local scale, global warming has favored the reproduction of urchins which acting in synergy with lobster fishing has reduced kelp resilience[3,4].

2. Comparison with other regime shifts and their CLDs

The previous example shows the structure of our data collection framework. Based on a review of the literature we collect information about how the regime shift works, the alternative regimes, important feedback processes underlying the shift, and reported drivers. All information is synthesized in both structured and unstructured text as well as a graphical representation of the system structure (CLD). Here we compare the CLD of Kelp transitions already introduced with another example from the database to exemplify how we deal with different direct and indirect drivers.

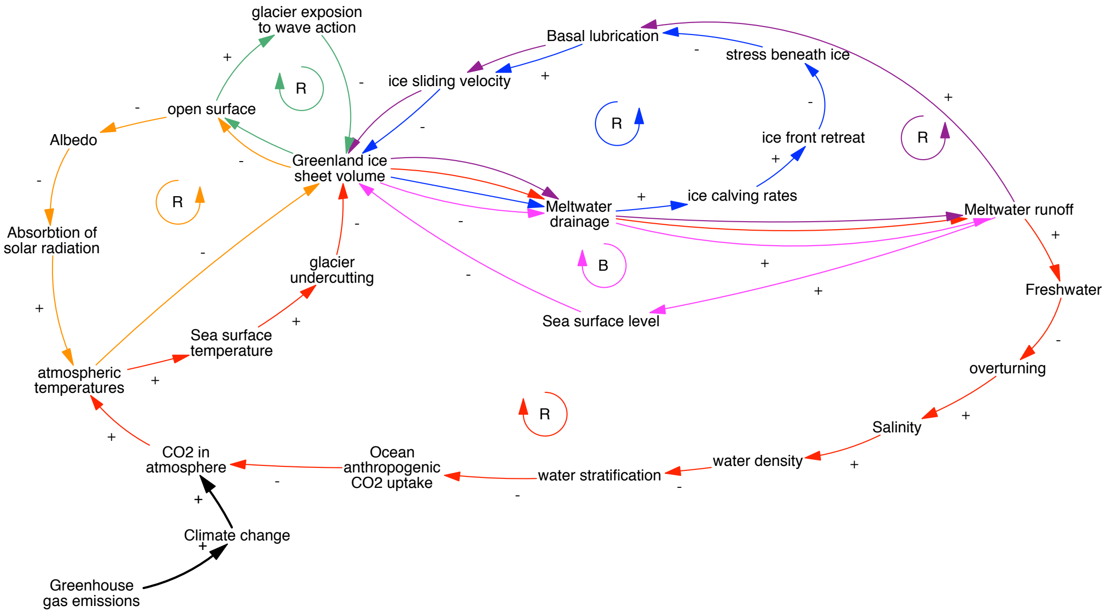

Figure 3. Causal loop diagram for Greenland Ice Sheet collapse. Developed by Rolands Sadawskis (2011, source: www.regimeshifts.org)

Direct drivers are defined as those that directly affect the feedback mechanism underlying the shift while indirect drivers only affect other direct/indirect drivers. In the kelp example, fishing and nutrient inputs are direct drivers while water stratification is an indirect driver. However, the position of the driver changes if the system boundaries change to capture the dynamics of other regime shifts. Below we present the CLD for Greenland Ice Sheet collapse (Fig 3), where water stratification is part of the red reinforcing feedback. For Greenland, climate change plays a role of direct driver and greenhouse gases is an indirect one. Both of them, however, appear as indirect drivers in the kelp CLD.

For the purpose of our network analysis, we included all drivers (direct, indirect or within a feedback) and mark its qualitative position in the network as link type (Fig 1 on paper), and quantitatively measure its directedness as the shortest path to feedback loops included in the statistical analysis of exponential random graph models (S2 Table).

Supplementary references

1. Steneck, R. *et al.* Kelp forest ecosystems: biodiversity, stability, resilience and

future. *Environ. Conserv.* **29,** 436–459 (2002).
2. Gorman, D. & Connell, S. Recovering subtidal forests in human-dominated landscapes. *J Appl Ecol* **46,** 1258–1265 (2009).
3. Ling, S., Johnson, C., Frusher, S. & Ridgway, K. Overfishing reduces resilience of kelp beds to climate-driven catastrophic phase shift. *P Natl Acad Sci Usa* **106,** 22341–22345 (2009).
4. Ling, S. D. *et al.* Global regime shift dynamics of catastrophic sea urchin overgrazing. *Philos. Trans. R. Soc. Lond., B, Biol. Sci.* **370,** 20130269–20130269 (2015).
5. Konar, B. & Estes, J. A. THE STABILITY OF BOUNDARY REGIONS BETWEEN KELP BEDS AND DEFORESTED AREAS. *Ecology* **84,** 174–185 (2003).
6. Steneck, R., Vavrinec, J. & Leland, A. Accelerating trophic-level dysfunction in kelp forest ecosystems of the western North Atlantic. *Ecosystems* **7,** 323–332 (2004).
7. Lauzon-Guay, J.-S., Scheibling, R. & Barbeau, M. Modelling phase shifts in a rocky subtidal ecosystem. *Mar Ecol-Prog Ser* **375,** 25–39 (2009).
8. Scheffer, M. *Critical Transitions in Nature and Society*. (Princeton University Press, 2009).
9. Gorman, D., Russell, B. D. & Connell, S. D. Land-to-sea connectivity: linking human-derived terrestrial subsidies to subtidal habitat change on open rocky coasts. *Ecol Appl* **19,** 1114–1126 (2009).
10. Pauly, D., Christensen, V., Dalsgaard, J., Froese, R. & Torres, F. Fishing down marine food webs. *Science* **279,** 860–863 (1998).
11. Estes, J., Terborgh, J., Brashares, J. & Power, M. Trophic Downgrading of Planet Earth. *Science* (2011).
12. Smith, V. H. & Schindler, D. W. Eutrophication science: where do we go from here? *Trends Ecol Evol* **24,** 201–207 (2009).
13. Bakun, A., FIELD, D. B., Redondo-Rodriguez, A. & WEEKS, S. J. Greenhouse gas, upwelling-favorable winds, and the future of coastal ocean upwelling ecosystems. *Glob Change Biol* **16,** 1213–1228 (2010).
