# Supplementary material for: Regime Shifts in the Anthropocene: Drivers, Risks, and Resilience
Source: PLoS One. 2015 Aug 12;10(8):e0134639. doi: 10.1371/journal.pone.0134639 (PMC4533971; doi:10.1371/journal.pone.0134639)
Supplement: S1 Table — On the left the list of 57 drivers identified which corresponds to the drivers displayed in Fig 1. To facilitate the interpretation of the results we classified drivers into 15 specific categories and 5 broader categories (Fig 2) inspired by existing classification of drivers in references10,25. Note that the broad categories are a simple aggregation of the specific ones given that specific categories are mutually exclusive. (DOCX) [file pone.0134639.s004.docx]

| **Drivers (N=57)** | **Specific categories (N=15)** | **Broad categories (N=5)** |
| --- | --- | --- |
| Agriculture | Crop and livestock production | Food Production |
| Aquaculture | Aquaculture | Food Production |
| Aquifers depletion | Ground water extraction | Resource Extraction |
| Climate change | Climate spillover | Spill over effects |
| Coastal erosion | Ecological spillovers | Spill over effects |
| Deforestation | Deforestation and fragmentation | Habitat Modification |
| Disease | Biological change | Habitat Modification |
| Droughts | Climate spillover | Spill over effects |
| ENSO like events | Climate spillover | Spill over effects |
| Erosion | Ecological spillovers | Spill over effects |
| Estuarine fresh water input | Ecological spillovers | Spill over effects |
| Estuarine salinity | Ecological spillovers | Spill over effects |
| Fertilizers use | Nutrients and pollution | Nutrients and Pollution |
| Fire frequency | Disturbance regimes | Habitat Modification |
| Fishing | Fisheries and marine harvest | Resource Extraction |
| Floods | Climate spillover | Spill over effects |
| Flushing | Ecological spillovers | Spill over effects |
| Green house gases | Green house gases | Nutrients and Pollution |
| Harvesting (animals) | Fisheries and marine harvest | Resource Extraction |
| Hunting | Hunting and logging | Resource Extraction |
| Ice melt water | Oceanic spillovers | Spill over effects |
| Impoundments | Infrastructure | Habitat Modification |
| Invasive species | Biological change | Habitat Modification |
| Irrigation | Infrastructure | Habitat Modification |
| Landscape fragmentation | Deforestation and fragmentation | Habitat Modification |
| Logging | Hunting and logging | Resource Extraction |
| Low tides | Oceanic spillovers | Spill over effects |
| Nutrient inputs | Nutrients and pollution | Nutrients and Pollution |
| Ocean acidification | Oceanic spillovers | Spill over effects |
| Pollutants | Nutrients and pollution | Nutrients and Pollution |
| Precipitation | Climate spillover | Spill over effects |
| Production intensification | Crop and livestock production | Food Production |
| Rainfall variability | Climate spillover | Spill over effects |
| Ranching (livestock) | Crop and livestock production | Food Production |
| River channelization | Infrastructure | Habitat Modification |
| Roads and railways | Infrastructure | Habitat Modification |
| Salt water intrusion | Ecological spillovers | Spill over effects |
| Sea level rise | Oceanic spillovers | Spill over effects |
| Sea surface temperature | Oceanic spillovers | Spill over effects |
| Sea water density | Oceanic spillovers | Spill over effects |
| Sediments | Ecological spillovers | Spill over effects |
| Sewage | Nutrients and pollution | Nutrients and Pollution |
| Soil moisture | Ecological spillovers | Spill over effects |
| Storms | Climate spillover | Spill over effects |
| Temperature | Climate spillover | Spill over effects |
| Thermal anomalies in summer | Climate spillover | Spill over effects |
| Turbidity | Ecological spillovers | Spill over effects |
| Upwellings | Oceanic spillovers | Spill over effects |
| Urban storm water runoff | Ecological spillovers | Spill over effects |
| Urbanization | Urbanization | Habitat Modification |
| Water depth | Ecological spillovers | Spill over effects |
| Water infrastructure | Infrastructure | Habitat Modification |
| Water level fluctuation | Ecological spillovers | Spill over effects |
| Water stratification | Ecological spillovers | Spill over effects |
| Water vapor | Climate spillover | Spill over effects |
| Wetland Drainage | Infrastructure | Habitat Modification |
| Wind stress | Climate spillover | Spill over effects |
