# Supplementary material for: Regime Shifts in the Anthropocene: Drivers, Risks, and Resilience
Source: PLoS One. 2015 Aug 12;10(8):e0134639. doi: 10.1371/journal.pone.0134639 (PMC4533971; doi:10.1371/journal.pone.0134639)
Supplement: S2 Table — Models 01 to 05 are null models following the specifications for bipartite networks31–34. Model 01 is a Markov random model. Model 02 explores the effect of 2 and 3 paths on both projections of the bipartite network (terms b1star2, b1star3, b2star2 and b2star3). Model 03 explore the effects of three-paths and cycles also known as clustering model. Model 04 is a curved exponential model that show the effects of geometrically weighted node shared partners (GWNSP), this is the number of open triangles that simultaneously share two basal nodes, thus a proxy for drivers or regime shifts co-occurrence. The weight of such number is adjusted with parameter alpha. Model 05 adds geometrically weighted terms for the degree (number of links) on each one-mode projection. Model 41 is the model that exhibited the best fit following both Akaike Information Criterion (AIC) and Maximum Likelihood Estimation (MLE). Model 41 combines a curved exponential model and explores the effects of homophily—the likelihood of two nodes of being connected on the one-mode projections given that they share attributes: scale of management for driver nodes, ecosystem type of regime shifts nodes, and nestedness and frequency as node covariates respectively. All model are dyadic dependent, only model 41 do not exhibit degeneracy. Significance levels: ***P<0.001, **P<0.01, *P<0.05, ·P< 0.1 (DOCX) [file pone.0134639.s005.docx]

| **Bipartite network models** | **Mod01** | **Mod02** | **Mod03** | **Mod04** | **Mod05** | **Mod41** |
| --- | --- | --- | --- | --- | --- | --- |
| Density | -1957*** | -6.50e+03*** | -4.00e+03*** | -467.193· | -4.88e+02*** | -2.80e+03· |
| b1star2 |  | 2.64e-01*** | 5.85e-01*** |  |  |  |
| b1star3 |  | 8.81e-03*** | 1.31e-02 |  |  |  |
| b2star2 |  | 2.33e-01*** | 3.94e-01*** |  |  |  |
| b2star3 |  | -2.77e-03 | -5.84e-03 |  |  |  |
| Three-paths |  |  | -5.07e-02 |  |  |  |
| Cycle-4 |  |  | 1.59e-01* |  |  |  |
| GWNSP |  |  |  | -0.230*** | 6.22e-02 | -1.18e-01 |
| gwnsp-alpha |  |  |  | 0.10629 | 1.679· | 1.45*** |
| gwb1deg0.5 |  |  |  |  | -8.22e-01 |  |
| gwb2deg0.5 |  |  |  |  | -1.84e+01*** |  |
| b1starmix.2 |  |  |  |  |  |  |
| Driver management: |  |  |  |  |  |  |
| global |  |  |  |  |  | 5.42e-02 |
| local |  |  |  |  |  | 1.33e-01** |
| regional |  |  |  |  |  | 1.01e-01· |
| b2starmix.2 |  |  |  |  |  |  |
| RegimeShift.Ecotype: |  |  |  |  |  |  |
| aquatic |  |  |  |  |  | -5.40e-03 |
| subcontinental |  |  |  |  |  | 2.11e-01*** |
| terrestrial |  |  |  |  |  | 2.20e-02 |
| Node covariates |  |  |  |  |  |  |
| Nestedness.Drivers |  |  |  |  |  | -7.43e-01 |
| Nestedness.RegimeShift |  |  |  |  |  | -1.32 |
| Frequency.Drivers |  |  |  |  |  | 3.75*** |
| Frequency.RS |  |  |  |  |  | 6.61* |
| **AIC** | 1436 | 13947 | 2261 | 1377 | 2082 | 1069 |
| **MLE** | -717.2127 (df=1) | -6968.571 (df=5) | -1123.731 (df=7) | -685.3822 (df=3) | -1035.953 (df=5) | -521.6868 (df=13) |
