# Supplementary material for: Regime Shifts in the Anthropocene: Drivers, Risks, and Resilience
Source: PLoS One. 2015 Aug 12;10(8):e0134639. doi: 10.1371/journal.pone.0134639 (PMC4533971; doi:10.1371/journal.pone.0134639)
Supplement: S3 Table — Models fitted for the one-mode projections have weighted links, therefore null models only count non-zero links and its sum37, taking as reference mode the Poisson distribution (Both Mod.RS.Null and Mod.D.Null). Two models were fitted on the regime shifts projection: Mod.RS.1 tested the effect of homophily (‘Nodematch’) on ecosystem type, this is whether the likelihood of two regime shifts sharing drivers is influenced by occurring on the same ecosystem type. The term ‘Nodefactor’ tested whether the likelihood is influenced by each of the ecosystem types taken as a factor for the regime shifts network. Node covariates was tested for nestedness, number of papers on the ISI web of science, and frequency. On the one-mode projections, frequency is measured as the number of links on the bipartite network over all possible number of links. The second model on the regime shifts network (Mod.RS.2) complemented the first by adding an extra set of terms that assessed the edge covariates with the information from the regime shifts database (RSDB). For the drivers network projection (Mod.D.1) homophily was assessed for a match on the driver’s scale of management and match on the driver’s categories (Fig 2). The effect of each variable as factors was assessed for the scale of management, and node covariates were tested for nestedness, directedness and frequency. The best models fitted were Mod.RS.2 for regime shifts and Mod.D.1 for drivers following both Akaike Information Criterion (AIC) and Maximum Likelihood Estimation (MLE). All model are dyadic dependent, and none of them exhibit degeneracy. Significance levels: ***P<0.001, **P<0.01, *P<0.05, ·P< 0.1 (DOCX) [file pone.0134639.s006.docx]

| **One-mode networks** | **Mod.RS.null** | **Mod.RS.1** | **Mod.RS.2** | **Mod.D. Null** | **Mod.D.1** |
| --- | --- | --- | --- | --- | --- |
| **Non zero** | 1.62*** | 0.12 | 0.28 | -1.87*** | -0.32*** |
| **Sum** | 1.37*** | -2.79*** | -2.73*** | 0.71*** | -0.98*** |
| **Nodematch.RS** |  |  |  |  |  |
| **Ecotype** |  |  |  |  |  |
| **Aquatic** |  | 0.82*** | 0.42* |  |  |
| **Subcontinental** |  | 0.74** | 0.45· |  |  |
| **Terrestrial** |  | 0.22 | 0.02 |  |  |
| **Nodematch.D** |  |  |  |  |  |
| **Driver Management** |  |  |  |  | 0.10* |
| **Driver Category** |  |  |  |  | 0.11 |
| **Nodefactor.RS** |  |  |  |  |  |
| **Ecotype.global** |  | 0.42** | 0.51** |  |  |
| **Ecotype.terrestrial** |  | 0.44** | 0.42* |  |  |
| **Nodefactor.D** |  |  |  |  |  |
| **Management local** |  |  |  |  | 0.18*** |
| **Management regional** |  |  |  |  | 0.14** |
| **Nodecov.RS** |  |  |  |  |  |
| **Nestedness** |  | 0.63** | 0.48· |  | -0.50*** |
| **Papers** |  | 0 | 0 |  |  |
| **Directedness** |  |  |  |  | -0.05 |
| **Frequency** |  | 6.24*** | 6.02*** |  | 2.76*** |
| **Edgecov - RSDB** |  |  |  |  |  |
| **Ecosystem process** |  |  | 0.52* |  |  |
| **Ecosystem service** |  |  | 1.50* |  |  |
| **Human well being** |  |  | 0.61 |  |  |
| **Ecosystem type** |  |  | 1.45· |  |  |
| **Land use** |  |  | 0.79 |  |  |
| **Scales** |  |  | 0.94* |  |  |
| **Reversibility** |  |  | 0.4* |  |  |
| **Evidence** |  |  | -0.34 |  |  |
| **Confidence of existence** |  |  | 0.12 |  |  |
| **Confidence of mechanism** |  |  | 0.05 |  |  |
| **AIC** | **-1250** | **-1694** | **-1715** | **-529.2** | **-1811** |
| **MLE** | **627.05**  **(df=2)** | **856.8**  **(df=10)** | **877.6**  **(df=20)** | **266.57**  **(df=2)** | **914.42**  **(df=9)** |
